# DCM-Related Tropomyosin Mutants E40K/E54K Over-Inhibit the Actomyosin Interaction and Lead to a Decrease in the Number of Cycling Cross-Bridges

**DOI:** 10.1371/journal.pone.0047471

**Published:** 2012-10-15

**Authors:** Fan Bai, Heather L. Groth, Masataka Kawai

**Affiliations:** Departments of Anatomy and Cell Biology, and Internal Medicine, University of Iowa, Iowa City, Iowa, United States of America; University of Minnesota, United States of America

## Abstract

Two DCM mutants (E40K and E54K) of tropomyosin (Tm) were examined using the thin-filament extraction/reconstitu­tion technique. The effects of the Ca^2+^, ATP, phos­phate (Pi), and ADP concentrations on isometric tension and its transients were studied at 25°C, and the results were com­pared to those for the WT protein. Our results indicate that both E40K and E54K have a significantly lower *T*_HC_ (high Ca^2+^ ten­sion at pCa 4.66) (E40K: 1.21±0.06 *T*_a_, ±SEM, N = 34; E54K: 1.24±0.07 *T*_a_, N = 28), a significantly lower *T*_LC_ (low- Ca^2+^ tension at pCa 7.0) (E40K: 0.07±0.02 *T*_a_, N = 34; E54K: 0.06±0.02 *T*_a_, N = 28), and a significantly lower *T*_act_ (Ca^2+^ activatable tension) (*T*_act_ = *T*_HC_–T_LC,_ E40K: 1.15±0.08 *T*_a_, N = 34; E54K: 1.18±0.06 *T*_a_, N = 28) than WT (*T*_HC_ = 1.53±0.07 *T*_a_, *T*_LC_ = 0.12±0.01 *T*_a_, *T*_act_ = 1.40±0.07 *T*_a_, N = 25). All tensions were normalized to *T*_a_ ( = 13.9±0.8 kPa, N = 57), the ten­sion of actin-filament reconstituted cardiac fibers (myocardium) under the standard activating conditions. The Ca^2+^ sensitivity (pCa_50_) of E40K (5.23±0.02, N = 34) and E54K (5.24±0.03, N = 28) was similar to that of the WT protein (5.26±0.03, N = 25). The cooper­a­tivity increased significantly in E54K (3.73±0.25, N = 28) compared to WT (2.80±0.17, N = 25). Seven kinetic constants were deduced using sinusoidal analysis at pCa 4.66. These results enabled us to calculate the cross-bridge distribution in the strongly attached states, and thereby deduce the force/cross-bridge. The results indicate that the force/cross-bridge is ∼15% less in E54K than WT, but remains similar to that of the WT protein in the case of E40K. We conclude that over-inhibition of the actomyosin interaction by E40K and E54K Tm mutants leads to a decreased force-generating ability at systole, which is the main mechanism underlying the early pathogenesis of DCM.

## Introduction

Dilated cardiomyopathy (DCM) is a myocardial disorder that leads to heart failure and sudden cardiac death [1]. In United States, DCM affects 30–40 people in 100,000 [2] and accounts for ∼10,000 deaths (∼10% of those affected) annually [3]. The main clinical manifestation of DCM is dilation of the left ventricle (LV) and systolic dysfunction. *In vitro* studies have suggested that the systolic dysfunction observed in DCM may be directly related to a decrease in the ATP hydrolysis rate [4].

Although the causes of DCM are diverse and thus general conclusions are often inappropriate, it is now clear that at least 25–30% of the DCM is caused by inherited genetic defects. Hence it is also called familial dilated cardiomyopathy (DCM) [5], [6]. Mutations in more than 20 genes, including several that encode sarcomeric proteins, have been implicated in DCM [7]. Unlike familial hypertrophic cardiomyopathy (FHC, HCM), where mutations in genes encoding sarcomeric proteins play a major role in disease pathogenesis, in the case of DCM the number of such mutations is small and they have not been studied extensively. So far, 12 α-tropomyosin (Tm) mutants are known to cause HCM, whereas only three Tm mutants (E40K, E54K and D230N) are known to cause DCM [8], [9].

Notably, different mutations within a single gene encoding a sarcomeric protein (actin, myosin, troponin, MyBP-C, etc.) can lead to one of two distinct diseases, HCM or DCM [10]. It would be interesting to learn why mutations within a single gene result in two different phenotypes. Previous studies have indicated that the pCa_50_ (Ca sensitivity, or [Ca^2+^] when tension reaches half of the maximum tension and defined by Eq. 1 in Methods) alteration caused by a particular mutation might be critical to emergence of a specific disease state: *in vitro* ATPase and motility assays have shown that HCM mutants increase pCa_50_, whereas DCM mutants decrease pCa_50_ [11]. However, in isometric tension studies, investigators also reported increased pCa_50_ in DCM myocardium [12], [13]. McConnell *et al.* have reported that in transgenic mice the pathogenesis, and thus disease phenotype, depend on the expression level of the mutant protein [14].

Since HCM generally results in diastolic dysfunction [15], and DCM generally results in systolic dysfunction [3], it has been hypothesized that a difference in myocardial contractility is the key determinant of a distinct disease phenotype. Also, the Potter’s group has shown that in DCM-associated Tn mutant mice, the decrease in the capacity for force generation is likely caused by reduced actomyosin binding [16]. Recently, we have reported that an HCM-associated Tm mutant caused elevated tension at low Ca^2+^ (pCa 8), from which we inferred that the molecular mechanism responsible is impaired relaxation [17]. Consequently, a tension-based study of the DCM-related mutants would be beneficial in investigating the difference between DCM and HCM pathogenesis.

Tm is a coiled-coil molecule stabilized by a heptad repeat: E40 (at the *e* position) of one chain interacts with R35 (*g*) of another, and E54 (*e*) of one chain interacts with K49 (*g*) of another, both through an electrostatic force [18], [19]. Also, it should be noted that E40 and E54 are located within the actin-binding motif of the Tm N-terminal region [20]. Mutants carrying an amino acid of the opposite charge may be destabilized, resulting in a change in the affinity of Tm for actin. Furthermore, E40K is located in period 1b, which interacts with actin in the presence of Ca^2+^, whereas E54K is located in period 2a, which interacts with actin in the absence of Ca^2+^ [21]. Previous studies have shown that the Ca^2+^ sensitivity of myocardium containing either of these mutants is decreased [22], and this is thought to be critical to DCM pathogenesis [11], [23]. At the same time, the maximum ATPase activity of E40K was shown to be lower than that of the WT protein [23]. A solution study performed in the absence of Tn showed that E40K reduced the cross-bridge turnover rate (decreased *K*_T_) and weakened the affinity of Tm for actin in the presence of myosin [23]. E54K showed only a weakened Tm-actin affinity in the absence of Tn and myosin (other parameters stayed unchanged from WT), leading to the hypothesis that E40K and E54K cause the DCM phenotype through different mechanisms [23].

The effect of a DCM-related Tm mutant on cross-bridge kinetics has never been investigated. Furthermore, most earlier studies did not include information on stiffness in the myocardial system under systolic and diastolic conditions. Here we report the effects of two DCM-related Tm mutants on isometric force, stiffness, and cross-bridge kinetics, as assessed using the thin-filament removal/reconstitution technique combined with sinusoidal analysis [24], [25], [26], and compared the results to those for WT Tm. Our results demonstrate that: 1) the systolic dysfunction in DCM is caused by decreased myocardial contractility, as indicated by the significant decrease in Ca^2+^-activatable tension in both mutants; 2) the pCa_50_ of the reconstituted myocardium does not change for either mutant in a solution mimicking the physiological composition; and 3) diastolic dysfunction in Tm mutation related DCM is caused by over-inhibition of the myocardium (based on a significant decrease in the number of cycling cross-bridges and reduced isometric tension at pCa 7.0).

## Materials and Methods

### Experimental Materials and the Thin-Filament Extraction/Reconstitution Technique

All animal studies conducted at the University of Iowa were in accordance with institutional guidelines and protocols approved by the Animal Care and Use Committee (ACUC). The University of Iowa has an Animal Welfare Assurance on file with the Office of Laboratory Animal Welfare (OLAW), National Institutes of Health. Research projects performed at Kawai’s laboratory using animals were approved by institutional ACUC.

Bovine cardiac muscles were dissected from trabeculae that were freely suspended in the right ventricle, split into small bundles, chemically skinned, and stored at −20°C [24]. These bundles can be stored for up to 2–3 months without altering their activity. On the day an experiment was carried out, these were further split into thin bundles (length ∼2 mm, diameter 90–110 µm). The latter were attached to the experimental apparatus using nail polish, and further skinned in 1% Triton X100 for 20 min in the relaxing solution (Table S1 in Ref. [17]). Preparations were then stretched to a sarcomere length of ∼2.1 µm, and the diameter and the length were measured. These values were used to calculate isometric tension, and the elastic and viscous moduli.

The preparations were then used for extraction, reconstitution, and subsequent mechanical measurements of the thin filament. The technique for extracting and reconstituting the thin filament was originally developed in the Ishiwata laboratory [26] and refined in the Kawai laboratory [25], and was performed as described [17]. Typical slow pen traces of tension time courses, light and electron micrographs at each stage of extraction and reconstitution, and associated SDS-PAGE were published previously [24], [25], [26], [27], [28]. In brief, the thin-filament (contains actin, tropomyosin, and troponin) was first extracted from skinned muscle fibers using gelsolin, a plasma protein which severs actin and thin filaments. The actin filament was then reconstituted in the fibers, in which active tension was measured and termed as *T*_a_. Then the regulatory proteins, Tm and Tn, were reconstituted in the fibers (this was when mutant Tm was used) to complete the reconstitution process.

### Proteins

G-actin was purified from rabbit fast-twitch skeletal muscle as described [29], and bovine cardiac Tn was purified from bovine hearts as described [30]. Both proteins were purified in the Kawai laboratory. Human WT and mutant α-Tms were expressed as recombinant proteins in *E. coli* and purified in the Dr. James D. Potter labortory. 100% pure mutant Tm was used in the reconstitution. These Tms have two extra amino acids, Ala-Ser, at the N-terminus, and these functionally substitute for acetylation [31]. Previous studies showed that this N-terminal extension is necessary for normal Tm function but has little effect on either protein stability [22] or Ca^2+^ sensitivity [32].

### Experimental Solutions

For the ATP, Pi and ADP studies, exactly the same solutions were used as previously reported (Table S1 in [17]). Two sets of solutions were used for the pCa studies. One set has been used for many years by us, is called “high ionic strength (IS) solutions”, and contains: 6 mM total of K_2_H_2_EGTA and K_2_CaEGTA, 6.1 mM Na_2_H_2_ATP, 6.6 mM MgAc_2_ (Ac = acetate), 8 mM K_1.5_H_1.5_Pi, 54 mM KAc, 3 mM NaAc, 10 mM NaN_3_, 10 mM MOPS, 15 mM creatine phosphate (Na_2_CP) and 320 units/ml creatine kinase (CK). The IS of this solution is 200 mM, [MgATP^2-^] is 5 mM, free [Mg^2+^] 1 mM, [Na^+^] 55 mM, and the pH is adjusted to 7.00 using KOH [17]. The other set was used by Chang and Potter in their study on E40K and E54K, is called “low-IS solution”, and contains: 7 mM total of K_2_H_2_EGTA and K_2_CaEGTA, 4.2 mM MgAc_2,_ 40 mM KAc, 2.5 mM Na_2_MgATP, no added Pi, 20 mM MOPS, 20 mM Na_2_CP, and 15 U/ml CK. The IS of this solution is 150 mM, [MgATP^2-^] is 2.2 mM, free [Mg^2+^] 1.3 mM, [Na^+^] 45 mM, and the pH is adjusted to 7.00 using KOH [22].

### pCa-Tension Study

The pCa-tension study was performed as described [32], in the range of pCa 7.0–4.0. The tension and stiffness at pCa 7.0 were called low Ca^2+^ tension (*T*_LC_) and low Ca^2+^ stiffness (*Y*_LC_). Here the stiffness is defined as *Y*_∞_ [33], but the ∞ symbol is dropped for simplicity. The tension and stiffness at pCa 4.0 were called high Ca^2+^ tension (*T*_HC_) and high Ca^2+^ stiffness (*Y*_HC_). The tension baseline was defined as that existing in the “super relaxing” solution (Table S1 in [17]) at 0°C, in the presence of 6 mM EGTA and 40 mM BDM. There is no significant tension or cross-bridge cycling under these conditions. Subsequent tension measurements were performed as the tension was incrementally increased from the baseline level. The pCa-tension relationship was studied in both the high-IS and low-IS pCa solutions. The results were fitted to the Hill equation:
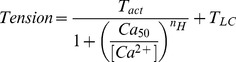
(1)where pCa = –log_10_[Ca^2+^], *T*_act_ is the Ca^2+^ activatable tension, and Ca_50_ is the apparent Ca^2+^ dissociation constant, which represents the Ca^2+^ concentration at half tension. pCa_50_ ( = –log_10_Ca_50_) represents Ca^2+^-sensitivity, and *n*_H_ (the Hill factor) represents the cooperativity. *T*_HC_ = *T*_act_+*T*_LC_ is the tension at high [Ca^2+^]. pCa-tension curves were individually fitted to Eq. 1, and the fitted parameters were averaged. All tension values were normalized to *T*_a_ of the actin-filament reconstituted myocardium without Tm or Tn, in the standard activating solution (5S8P, Table S2 in [17]). *T*_a_ averaged to 13.9±0.8 KPa (N = 57). All experiments were carried out at 25°C.

### Sinusoidal Analysis

**Figure 1:**
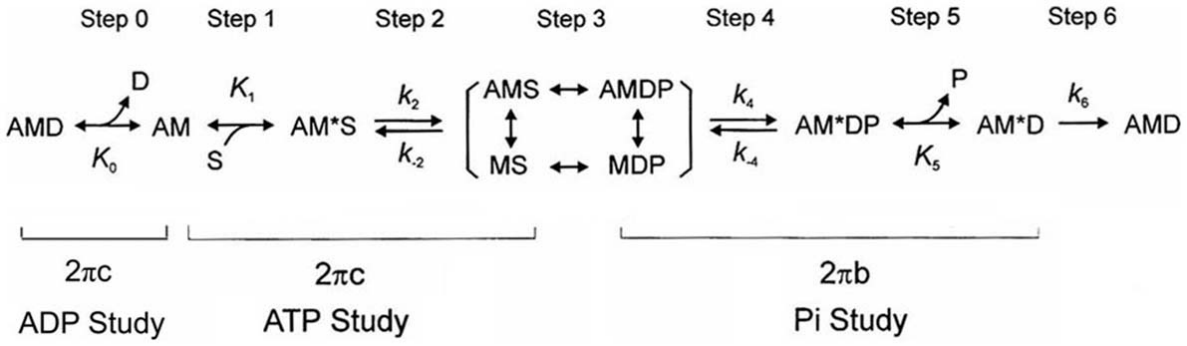
Elementary steps of the cross-bridge cycle. The uppercase letters *K* indicate the association or equilibrium constants, and the lowercase letters *k* the rate constants of the elementary steps. Collectively these are referred to as the “kinetic constants”. A = actin, M = Myosin, D = MgADP, S = MgATP, and P = Pi = Phosphate.

The elementary steps of the cross-bridge cycle based on six states (Fig. 1) were characterized by sinusoidal analysis performed as described [33], [34] in sets of solutions with 200 mM IS. Sinusoidal length changes of small amplitude (0.125% L_0_) were applied to the reconstituted myocardium at 18 different frequencies (*f*) in the range of 0.13 Hz to 100 Hz. The resulting tension transients were analyzed and the complex modulus *Y*(*f*) was calculated. *Y*(*f*) is the ratio of the stress change to the strain change represented in the frequency domain. *Y*(*f*) was fitted to Eq. 2, which incorporates 2 exponential processes [33]:

Process B Process C
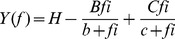
(2)where 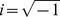; 2*πb* and 2*πc* (*b*<*c*) are the apparent rate constants of exponential processes B and C, respectively; *B* and *C* are their respective magnitudes (amplitudes), and *H* is a constant. *Y*_∞_ = *H*−*B*+*C* is the modulus extrapolated to the infinite frequency; all of these are real numbers. *B*, *C*, *H, and Y*_∞_ have the same units as *Y*(*f*) and isometric tension, and thus are normalized to *T*_a_. In cardiac muscle fibers, process A was not observed at ≤25°C [25], [34], [35]. The *Y*(*f*) thus measured includes the effect of series compliance, which may [36] or may not [37] affect the apparent rate constants. However, the series compliance is not a concern in this report because it does not differ between the mutant and WT proteins.

The apparent rate constants 2*πb* and 2*πc* were studied as functions of the MgATP (*S*), Pi (*P*) and MgADP (*D*) concentrations. The data were fitted to the following equations, which were derived from the cross-bridge scheme in Fig. 1, assuming that steps 0, 1 and 5 are in fast equilibrium, and that step 6 is the slowest forward step of the cross-bridge cycle [38].
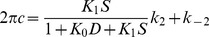
(3)

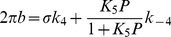
(4)where



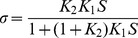
(5)
*K*_1_ is the ATP association constant, *K*_0_ is the ADP association constant, *k*_2_ is the forward rate constant of cross-bridge detachment step 2, and *k*_−2_ is its reversal step. *k*_4_ is the forward rate constant of the force generation (isomerization) step 4, and *k*_−4_ is its reversal step. *K*_5_ is the Pi association constant. *σ* in Eq. 5 was calculated from *K*_1_ and *K*_2_ obtained from the MgATP study and *S* = 5 mM, the condition of the Pi study. The reconstituted myocardium was studied at 8 different [MgATP] (0.05, 0.1, 0.2, 0.5, 1, 2, 5, 10 mM) with a fixed [Pi] (8 mM) and pCa 4.66, and the data were fitted to Eq. 3 to deduce the rate and association constants of steps 1 and 2. *D* = 0 was assumed in this study because of the presence of CP and CK. The effect of phosphate (Pi) was studied at 6 different [Pi] (0, 2, 4, 8, 16, 32 mM) with a fixed [MgATP] (5 mM) and pCa 4.66, and the data were fitted to Eq. 4 to deduce the rate and association constants of steps 4 and 5. The effect of MgADP was studied at 4 different [MgADP] (0, 1, 2, 3 mM), with fixed [Pi] (8 mM) and [MgATP] (2 mM), at pCa 4.66, and the data were fitted to Eq. 3 to deduce *K*_0_.

## Results

### pCa-tension and pCa-stiffness Studies

**Figure 2:**
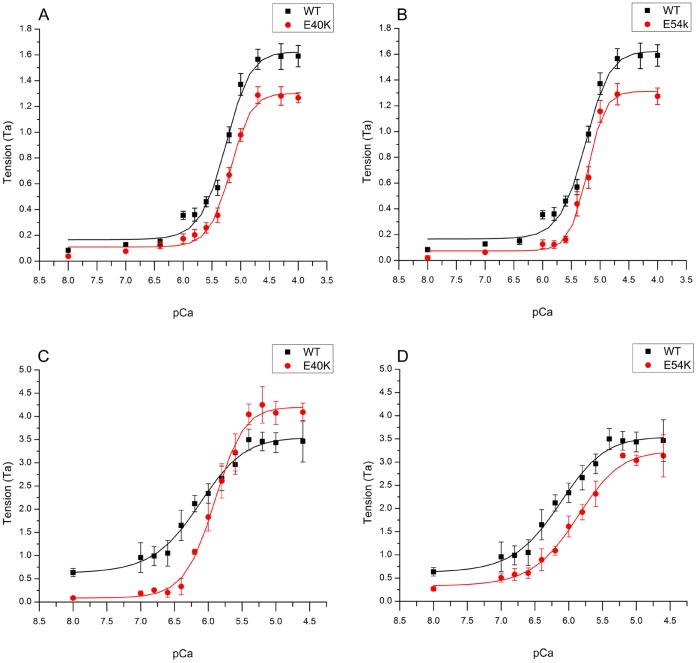
pCa-tension plots. (A, B) pCa-tension plots comparing mutant Tms and WT-Tm in high-IS solution. N = 25 for WT Tm; N = 34 for E40K; N = 28 for E54K. (C, D) pCa-tension plots comparing mutant Tms and WT-Tm in low-IS solution [22]. N = 8 for WT Tm; N = 10 for E40K; N = 9 for E54K. The means and their SEMs are shown. Curved lines are calculated from Eq. 1, based on best-fit parameters. Tension is normalized to that of actin-filament reconstituted fibers (T_a_ = 13.9±0.8 kPa, N = 57).

**Figure 3:**
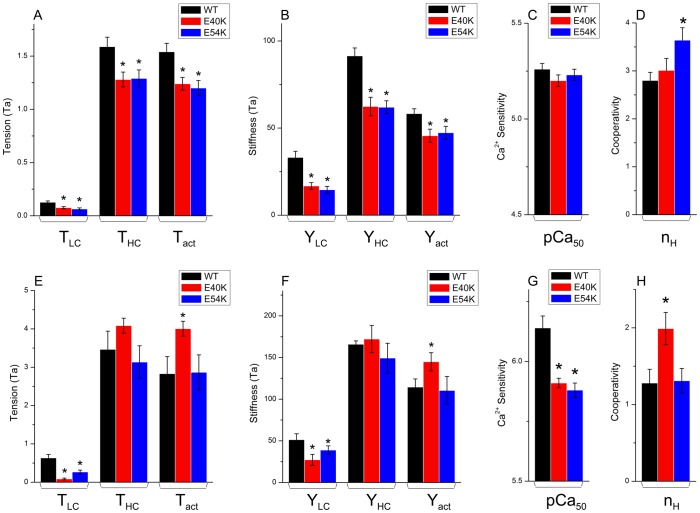
Summary of Tension (A and E), Stiffness (B and F), pCa_50_ (C and G), and Cooperativity (D and H). (A–D) Data obtained in high-IS solutions. (E–H) Data obtained in low-IS solutions [22]. (A, E) Low calcium tension (*T*_LC_) represents the tension at pCa 7. High calcium tension (*T*_HC_) represents the tension at pCa 4.66. Active tension (*T*_act_) is the tension developed on Ca^2+^ activation (*T*_act_ = *T*_HC_–*T*_LC_). (B, F) Results of the stiffness study. Tension and stiffness were normalized to Ta. (C, G) pCa_50_ (Ca^2+^ sensitivity). (D, H) Cooperativity. *: p<0.05.

To determine the effects of Tm mutants E40K and E54K on Ca^2+^-sensitivity and cooperativity, tension and stiffness of the thin filament-reconstituted myocardium were studied as functions of [Ca^2+^]. The pCa-tension plots comparing mutant and WT Tms are displayed in Fig. 2. Their parameters, as fitted to Eq. 1, are plotted in Fig. 3. As seen in these figures, both mutants affected the pCa-tension relationship. They also both showed significantly decreased *T*_HC_ (WT: 1.59±0.09 *T*_a_, N = 25; E40K: 1.28±0.07 *T*_a_, N = 34; E54K: 1.29±0.08 *T*_a_, N = 28), *T*_act_ (WT: 1.54±0.08 *T*_a_, N = 25; E40K: 1.24±0.06 *T*_a_, N = 34; E54K: 1.20±0.07 *T*_a_, N = 28) and *T*_LC_ (WT: 0.13±0.01 *T*_a_, N = 25; E40K: 0.08±0.01 *T*_a_, N = 34; E54K: 0.06±0.01 *T*_a_, N = 28) compared to WT (P<0.005), indicating that contractility was decreased during systole (*T*_HC_) and over-inhibited during diastole (*T*_LC_). The trend was the same for stiffness (Fig. 3B). The pCa_50_ of the E40K (5.20±0.03, N = 34, p = 0.25) or E54K (5.23±0.03, N = 28, p = 0.36)-reconstituted myocardium was not significantly different from that of WT (5.26±0.03, N = 25), demonstrating that Ca^2+^-sensitivity was unaltered (Fig. 3C). The cooperativity (*n*_H_) of E40K was similar to that of WT, whereas that of E54K was significantly greater than that of WT (Fig. 3D) (WT 2.80±0.17, E40K 3.01±0.25, E54K 3.64±0.26; see also Table S1 in [17]).

### pCa-tension Study using the Chang and Potter pCa Solution

Independent studies have indicated that both E40K and E54K cause a decrease in Ca^2+^ sensitivity, based on an ATPase assay, skinned fiber force measurement, and an *in vitro* motility assay [22], [39], [40]. Although the experimental conditions varied among these studies, the pCa solutions used for the ATPase assay and tension measurement analysis shared two common features: 150 mM ionic strength (IS) and no added Pi [22], [40]. These conditions are significantly different from those of our study: we used 200 mM IS and 8 mM Pi (high-IS solution, see Methods). To investigate the possible effects of these differences on the pCa_50_, we studied the pCa-tension curve of the reconstituted myocardium using the solution developed by Chang and Potter [22] (low IS solution, Methods). The result was also fitted to the Hill equation (Eq. 1).

In the low-IS solution, *T*_HC_ was generally 2–3x that of the high-IS solution (Fig. 3E vs. 3A). In general, the trend was not the same as that in the high-IS solution (Fig. 3E–H vs. 3A–D). E40K and E54K caused a significant decrease in pCa_50_ (E40K: 5.91±0.02, N = 10; E54K: 5.88±0.03, N = 9) compared to WT (6.14±0.05, N = 8) (Fig. 3G). E40K and E54K caused a significant decrease in *T*_LC_ (E40K: 0.08±0.02 *T*_a_, N = 10; E54K: 0.27±0.05 *T*_a_, N = 9) compared to WT (0.63±0.09 *T*_a_, N = 8). However, the data for *T*_HC_ were more scattered and the average values did not differ significantly among the mutant- and WT-reconstituted preparations (WT: 3.46±0.47 *T*_a_, N = 8; E40K: 4.08±0.2 *T*_a_, N = 10; E54K: 3.13±0.42 *T*_a_, N = 9). *T*_act_ was significantly increased in E40K (4.00±0.19 *T*_a_, N = 10), but *T*_act_ of E54K (2.87±0.45 *T*_a_, N = 9) remained similar to that in WT (2.83±0.45 *T*_a_, N = 8). The cooperativity remained similar between E54K (1.31±0.16, N = 9) and WT (1.28±0.18, N = 8), but increased significantly in E40K (1.99±0.21, N = 10) (Fig. 3H).

### Sinusoidal Analysis and Cross-bridge Kinetics

**Figure 4:**
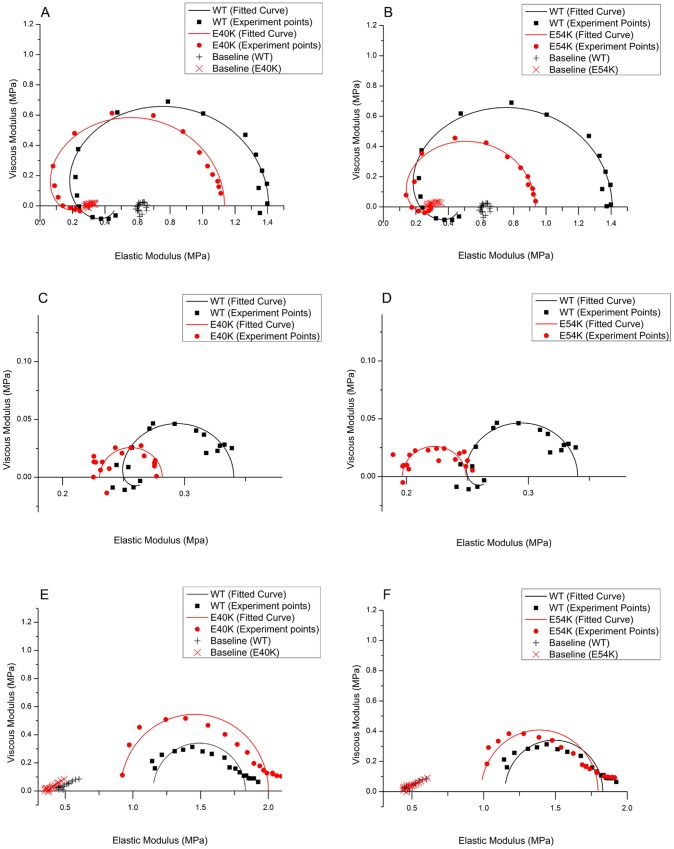
Nyquist plots of the mutants (red) compared to WT (black). (A–B) Two mutants are compared to WT at pCa 4.66; (C–D) Two mutants are compared to WT at pCa 7.0. Also included in A and B are the baselines taken in the relaxing solution at 0°C (**×** for the mutant, and **+** for WT). The data in A–D were obtained in high-IS solutions. (E–F) Two mutants are compared to WT at pCa 4.66 in low-IS solution [22]. Range of frequency used: 0.13–100 Hz. The frequency increases in the clock-wise direction. Note the difference in scale for both axes in panels A–B vs. panels C–D, and panels A–B vs. panels E–F.

Sinusoidal analysis was performed as described [25], [33] to assess cross-bridge kinetics and to characterize the elementary steps of the cross-bridge cycle. Nyquist plots comparing data for mutant (open symbols and dashed lines) and WT (filled symbols and solid lines) Tms are shown in Fig. 4, together with curves fitted to Eq. 2: panels A and B show Nyquist plots at pCa 4.66 under the standard activating condition (5S8P, Table S2 in [17]). Also shown are baseline records (+, ×) under the super-relaxing condition (Table S2 in [17]). Panels C and D show the data obtained at pCa 7. The Nyquist plot for the WT fibers demonstrates that an appreciable fraction of cross-bridges actively cycled and performed energy transduction, even at pCa 7 (black squares in Fig. 4C and D), as evidenced by the presence of processes B and C (represented by two contiguous semicircles), and as reported earlier for the myocardium [41]. However, in the cases of E40K and E54K, the magnitude of process C is significantly diminished, to ¼∼½, and process B is very small (*B* is close to 0) (red squares in Fig. 4C and D), indicating that energy transduction is significantly reduced; this result is consistent with the decreased *T*_LC_ (Fig. 3A) of these two mutants. The diameters of the semicircles are proportional to the number of actively cycling cross-bridges [38]. The data in Fig. 4C and D demonstrate that, in the case of WT, only ∼13% of the cross-bridges at pCa 4.66 are active at pCa 7.0. In both E40K and E54K, the diameter of the Nyquist plots was significantly smaller than that for WT, demonstrating that only 5–7% of cross-bridges cycle actively at pCa 7. We conclude, therefore, that the regulatory proteins over-inhibit the actomyosin interaction, and that this results in a decrease in *T*_LC_ and decreases in the magnitudes of *B* and *C* in both mutants at pCa 7.

The Nyquist plots for E40K and E54K in low-IS solution (pCa 4.66) are shown in Fig. 4E and F. It is clear that process B is absent (*B* = 0) when the low-IS solution is used; this is primarily due to the absence of Pi in the activating solution [38].

**Figure 5:**
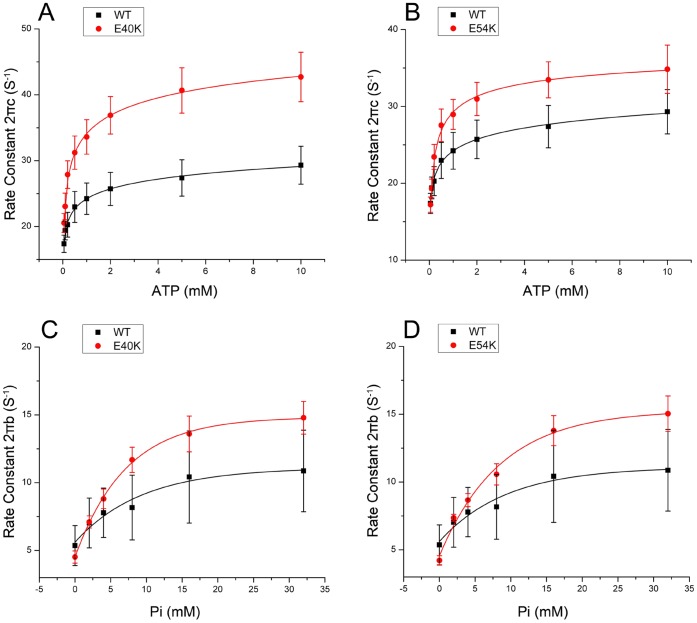
Apparent rate constants. The apparent rate constants for myocardium reconstituted with mutant Tms (red) and WT Tm (black). Symbols represent the mean±SEM. Continuous curves were generated by fitting the data to Eq. 3 (A and B) or Eq. 4 (C and D). (A–B): 2πc (in s^−1^) is plotted against [MgATP] (mM). (C–D): 2πb (in s^−1^) is plotted against [Pi] (mM).

**Figure 6:**
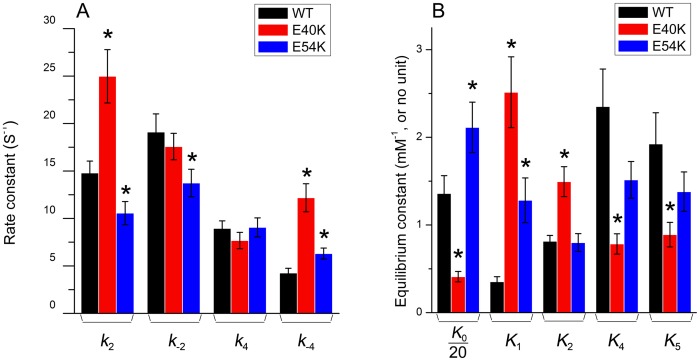
Kinetic constants. The kinetic constants of the cross-bridge cycle (Scheme 1) in reconstituted cardiac fibers are compared among mutant and WT Tms. (A) Rate constants. (B) Equili­brium constants. Note that *K*_0_ was divided by 20 and *K*_5_ was multiplied by 5. *: p<0.05.

Two apparent rate constants, 2*πb* and 2*πc*, were measured as functions of [MgATP] (Fig. 5A and B) and [Pi] (Fig. 5C and D) at pCa 4.66. E40K caused a significant increase in 2*πc* (Fig. 5A) and a small increase in 2*πb* (Fig. 5C) compared to WT. E54K also caused increases in these rate constants (Fig. 5B and D), but these were not significant. The rate and association constants (together called “kinetic constants”) of the elementary steps were deduced by fitting the ligand concentration dependence of the apparent rate constants to Eqs. 3 and 4 of [38]. The kinetic constants of the mutants and WT are compared in Fig. 6. All kinetic constants were changed in the mutants, but only the following changes were significant (p<0.05) compared to WT. E40K exhibited an ∼70% decrease in *K*_0_, an ∼610% increase in *K*_1_, an ∼80% increase in *K*_2_, an ∼70% decrease in *K*_4_, an ∼50% decrease in *K*_5_, and an ∼70% increase in *k*_2_, and an ∼190% increase in *k*_-4_. E54K exhibited an ∼60% increase in *K*_0_, an ∼260% increase in *K*_1_, an ∼29% decrease in *k*_2_, an ∼28% decrease in *k*_-2_, and an ∼50% increase in *k*_-4_. Interestingly, *k*_4_ (the rate constant of the force generating step) did not change in either mutant.

### Cross-bridge Distribution and Force per Cross-bridge

**Figure 7:**
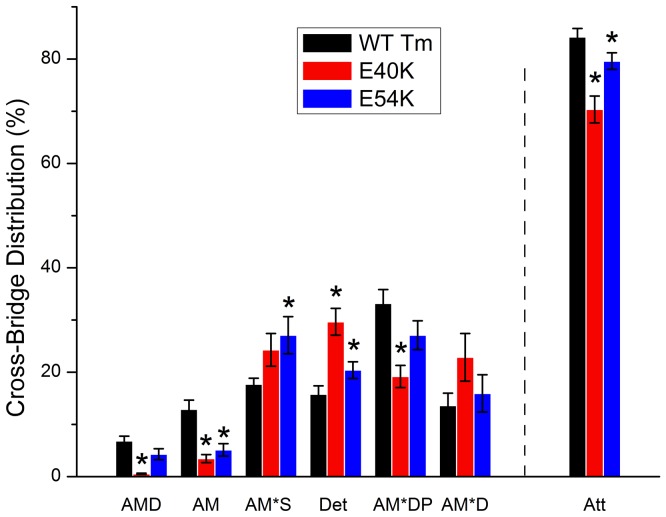
Cross-bridge distributions. From the equili­brium constants, the cross-bridge distribution over six states under the standard activating condition (5S8P, Table S2 in [17]) was calculated for two mutant and the WT Tm forms. Att indicates the sum of all strongly attached (force generating) cross-bridges: Att = AMD+AM+AM*S+AM*DP+AM*D.

There are two possible mechanisms through which the observed decrease in isometric tension during Ca^2+^ activation (*T*_HC_, Fig. 3A) can be explained: either the number of force-generating cross-bridges, or the force generated by each cross-bridge is decreased. To find out which one of these mechanisms is at work, we calculated the distribution of cross-bridges in each state under the standard activating condition [42]; the result is shown in Fig. 7. Both mutants showed a significant decrease in the AM state (E40K: ∼74%; E54K: ∼60%, p<0.05) and an increase in the Det state (E40K: ∼88%; E54K: ∼30%, p<0.05) compared to WT. The *Det* state includes weakly attached states (AMS and AMDP) and truly detached states (MS and MDP) (Fig. 1). E40K showed an ∼40% decrease in AM*DP, and E54K showed an ∼53% increase in AM*S compared to WT (p<0.05). Att indicates the sum of all strongly attached (force generating) cross-bridges: Att = AMD+AM+AM*S+AM*DP+AM*D = 1–Det. Both mutants showed a significantly decreased Att state (E40K: ∼17%; E54K: ∼6%) compared to WT, indicating that the number of force-generating cross-bridges is decreased. Force per cross-bridge was obtained as *T*_HC_/X_att_, which remained similar to that of WT in E40K, but decreased by ∼13±6% in E54K (with error propagation) compared to WT.

The values of tension (*T*_a_ = 13.9 kPa) produced in actin-filament reconstituted fibers in the current study are less than those reported in previous studies (eg: [43], [44], 39–75 kPa). This is because regulatory proteins, Tm and Tn, were absent when *T*_a_ was measured. Furthermore, 8 mM Pi and 200 mM ionic strength were used in our standard activating solution and the temperature at which the experiments was carried out was 25°C. In many previous experiments, no Pi was added, an ionic strength of 150 mM was often used, and some experiments were carried out at higher temperatures such as 37°C. In addition, these experiments were carried out in the presence of regulatory proteins. An addition of the regulatory proteins increases tension to 1.5×[45], [46]. The deletion of 8 mM Pi increases tension to 2×[17], [25], [47], and a decrease of ionic strength from 200 mM to 150 mM increases tension to 1.2×[48], [49]. See also Fig. 3A vs. 3E. An increase in temperature from 25°C to 37°C increases tension to 1.2–1.5 [46], [50], [51], [52]. When we consider the effects of regulatory proteins and Pi alone, the net effect is 3×( = 1.5×2), therefore 3*T*_a_ = 42 kPa, which compares reasonably well with other reports [43], [44]. The solution used in this study is more physiological condition, because physiological [P_i_] in contracting cardiomyocytes was reported to be ∼6 mM [53], and the ionic strength ∼215 mM [54].

**Table 1:** Comparison of HCM- and DCM-related Tm mutations on isomeric tension and cross-bridge kinetics.

Parameters (unit)	HCM mutants (V95A, D175N and E180G)	DCM mutants (E40K and E54K)
*T*_LC_ (T_a_)	All ↑ (2–3 fold)	Both ↓ (∼50%)
*T*_HC_ (T_a_)	E180G ↑ (∼17%)	Both ↓ (∼20%)
*T*_act_ (T_a_)	V95A and D175N ↓ (∼28%)	Both ↓ (∼20%)
*Y*_LC_ (T_a_)	All ↑ (2–2.5 fold)	Both ↓ (∼5%)
*Y*_HC_ (T_a_)	E180G ↑ (∼33%)	Both ↓ (∼33%)
*Y*_act_ (T_a_)	D175N ↓ (∼33%)	Both ↓ (∼20%)
pCa_50_	V95A and E180G ↑	Remain similar in high-IS solution Both ↓ in low-IS solution
Hill Factor (Cooperativity)	All ↓ (∼38%)	E54K ↑ (∼20%)
*K*_0_ (mM^−1^)	V95A↓ (∼40%) and E180G ↑ (∼35%)	E40K ↓ (∼70%)
*K*_1_ (mM^−1^)	V95A↓ (∼50%)	E40K ↑ (∼6 fold) E54K ↑ (∼3 fold)
*K*_2_	V95A↓ (∼33%)	E40K ↑ (∼84%)
*K*_4_	All remain similar to WT	E40K ↓ (∼67%)
*K*_5_ (mM^−1^)	V95A and D175N ↓ (∼60%)	E40K ↓ (∼50%)
*k*_2_ (s^−1^)	D175N ↑ (∼30%)	E40K ↑ (∼70%) E54K ↓ (∼30%)
*k_-_*_2_ (s^−1^)	V95A ↑ (∼60%)	E54K ↓ (∼20%)
*k*_ 4_ (s^−1^)	V95A ↑ (∼40%)	Both remain similar to WT
*k*_-4_ (s^−1^)	All remain similar to WT	E40K ↑ (∼2 fold), E54K ↑ (∼50%)

Notes: ↑ increase, ↓ decrease; Only significant changes are indicated; All percent change is in comparison to WT. The data on HCM are from earlier publication [17].

## Discussion

The clinical manifestations of DCM are highly diverse and caused by both genetic and non-genetic factors [55]. Mutations within 33 genes are known to cause DCM, and 23 of these (including all sarcomeric proteins and 5 of the Z-line proteins) are involved in the energy transduction processes [56]. Therefore, it is reasonable to postulate that the molecular pathogenesis of familial DCM is associated with deficits in myocardium contractility. Recent studies support the idea that sarcomeres are a key component of contraction and its regulation, rather than a simple assembly of force generators [57], [58], [59], [60]. The functional and regulatory properties of cardiac sarcomeres have been shown to be responsible for many of the mechanical properties of the heart [61], [62], [63], [64]. Tm is critical for maintaining normal function of the heart, as it is an essential element of the excitation-contraction coupling process and the thin-filament regulatory mechanism. In this report, we investigated early consequences of gene alterations of Tm, which eventually lead to DCM.

Compared to transgenic studies, our method has two advantages. 1) Transgenic studies are subject to significant secondary effects such as cardiac remodeling [65], myocyte disarray [66], interstitial fibrosis [67], altered post translational modifications including phosphorylation and dephosphorylation [68], and a loss of cardiac tissue [69], making it difficult to sort out the primary cause of pathogenesis. In contrast, our study characterizes the direct effect of the mutations on myocardial function and generates key information on the early pathogenesis of DCM. 2) It is difficult to control the expression levels of mutant proteins in transgenic studies. Our method ensures nearly 100% replacement with mutant proteins, as demonstrated by: SDS-PAGE [25], [26], [28], [45]; electron microscopy [24], [26], [28]; light microscopy [24], [26], [45]; mechanical tension [25], [26], [28], [45]; rate constant measurements [25], [28], [45], which reproduced 107±4% of isometric tension, 98±6% of rate constant 2 *Π b* and 92±4% of the rate constant 2 *Π c* (reviewed by [24]). Also, perfect reproduction of Ca^2+^ sensitivity and cooperativity has been reported [26].

In the present pCa-tension study, E40K and E54K showed a Ca^2+^ sensitivity similar to that of WT, in the presence of 8 mM Pi in high-IS solution. However, in the absence of added Pi in low-IS solution, the Ca^2+^ sensitivity decreased. The latter results are similar to those obtained previously [22], [23], [40]. It has been reported, based on studies using rabbit psoas fibers, that elevating IS from 128 mM to 201 mM decreases pCa_50_ by about 0.35 units [70], and that an increase in [Pi] from 0 to 7.5 mM decreases pCa_50_ by 0.23 units [71]. Other researchers reported a similar effect of [Pi] on pCa_50_ in cardiac fibers [47], [72]. The physiological concentration of ATP is reported to be ∼4.5 mM, and Pi to be ∼6 mM [53], [73]. The IS under physiological conditions may be as large as 215 mM [54]. It is apparent that our high IS solution is a better match to the physiological conditions.

E40K and E54K also showed similar *T*_HC_ to that of the WT in the low IS solution, while these mutants significantly decreased *T*_HC_ in the high IS. This is probably due to the dramatically increased number of strongly attached cross-bridges when lowering [Pi] and ionic strength [74], [75]. Sinusoidal analysis showed that E40K maintained similar T_HC_/(cross-bridge) with that of the WT and caused a ∼17% decrease in the number of actively cycling cross-bridges (decreased Att). E54K showed a ∼13% decrease in T_HC_/(cross-bridge) and a ∼6% decrease in Att. However, these changes are much smaller when compared with the effect of [Pi] and IS. The reconstituted fibers generated about 2.5×tension in low IS solution than in high IS solution (Fig. 3E vs. 3A). Therefore, the effect of mutations on cardiac contractility may not be as evident in the low IS solution as in the high IS solution.

Because previous studies have indicated that DCM-related mutations in sarcomeric proteins are associated with decreased pCa_50_ [11], it has been thought that decreased pCa_50_ may be the critical determinant of DCM pathogenesis. However, a recent transgenic study has indicated that familial DCM is not always associated with decreased pCa_50_ [76], and some earlier studies even reported increased pCa_50_ in the DCM myocardium [12], [13]. It also has been reported that Ca^2+^ sensitivity of the myocardium can be altered by other non-genetic factors such as remodeling [77] and exercise [78] without causing DCM or HCM. The results we report here also indicate that decreased pCa_50_ may not be the key molecular cause of DCM pathogenesis at the physiological Pi concentration and ionic strength (Fig. 3C). Therefore, we conclude that for the Tm E40K and E54K mutants, changes in pCa_50_ within muscle fibers may not be the key factor in disease pathogenesis.

A decrease in contractility may be the main cause for the systolic dysfunction in the context of DCM-related Tm mutants. Both E40K and E54K showed significantly decreased *T*_HC_ and *T*_act_ in the reconstituted myocardium (Fig. 3A), which could well explain the systolic dysfunction in the DCM heart. At the molecular level, our results indicate that the number of strongly attached cross-bridges decreased in both mutants (Fig. 7); in addition, force/cross-bridge was decreased in E54K. This is consistent with previous findings that DCM-related Tm mutations may decrease the proportion of strongly bound actomyosin complexes [79]. Recent studies in transgenic mice have shown that the E54K mutant caused a decrease in maximum tension in addition to a series of typical DCM phenotypes [40], suggesting that the decreased myocardial contractility may be the direct trigger of subsequent clinical manifestations, including dilation of the LV and deformation of the extracellular matrix. Therefore, cardiac remodeling events that lead to defects such as myocyte disarray and enlargement of the heart may be maladaptive compensation for the decrease in contractility.

The decrease in *T*_LC_ caused by E40K and E54K may contribute to the filling pattern in DCM patients, which is defined using the ratio *E*/*A* [80], where *E* represents the degree of filling due to relaxation of the LV, and *A* the degree of filling due to contraction of the left atrium (LA). The decrease in *T*_LC_ in DCM lowers the LV diastolic pressure, increases the early ventricular filling velocity, and results in an increase in the *E*/*A* ratio. Cardiac muscle relaxation is an energy consuming process [81]. The diminished process B (diminished magnitude *B*) of the Nyquist plot at pCa 7.0 (Fig. 4C and D) implies a diminished energy output during the early stages of diastole. We previously studied three HCM related Tm mutants (V95A, D175N, and E180G) [17] using the same techniques as in this report. Table 1 compares the effects of HCM- and DCM-related Tm mutants on isometric tension and cross-bridge kinetics, demonstrating that the effects are diverse and complex. These results suggest that the underlying molecular mechanism may be different for each mutant, and that they must be dealt with individually.

E40K and E54K are both located at the “e” position of the heptad repeat and respectively interact with R35 and K49 of another chain of Tm molecule to stabilize the coiled-coil structure [19]. The opposite charge (from E to K) introduced by these mutants can destabilize the local coiled-coil structure. Previous differential scanning calorimetry (DSC) study has shown that both E40K and E54K caused a significant loss of the stability in Tm [23]. It has been suggested that the destabilization of Tm’s coiled-coil structure promotes the Tm-actin binding [82]. Therefore, a likely scenario is that E40K and E54K enhance the Tm-actin binding to over-inhibit the actin-myosin interaction.

One generalization we can make is that Tm mutation-related HCM causes an elevation in *T*_LC_, whereas Tm mutation related DCM causes a decrease in *T*_LC_. These results demonstrate regulatory dysfunction in two opposite directions. Elevated *T*_LC_ observed in HCM may be directly responsible for the impaired relaxation and may lead to the disease phenotype [17]. Decreased *T*_LC_ is a sign that mutant Tm over-inhibits the actomyosin interaction in the absence of Ca^2+^, as suggested by a decrease in the number of actively cycling cross-bridges (Fig. 3B, decreased *Y*_LC_). This over-inhibition effect is also evidenced at pCa 4.66 (Fig. 3B, decreased *Y*_HC_; Fig. 7, decreased Att) and causes a decrease in *T*_HC_ (Fig. 3A).

The “three state model” proposed by McKillop and Geeves [83] may explain this over-inhibition effect. In the absence of Ca^2+^, enhanced binding of Tm and actin at the “blocked state” leads to an inadequate exposure of actin to myosin for interaction, leading to decreased *T*_LC_. In the presence of Ca^2+^, Tm azimuthally moves ∼25° to allow a moderate actin-myosin binding (closed state), then Tm moves further ∼5° to induce the strong actin-myosin binding (open state) [83]. Due to the enhanced Tm-actin binding, the two mutants are likely to shift the balance among the three states towards the “blocked state” and the “closed state”, thereby reducing the number of actively cycling cross-bridges at the “open state” to result in a decreased *T*_HC_.

At the same time, these two adjacent and similar mutants exhibited differences in force generation and cross-bridge kinetics. The most notable difference was that E54K decreased *T*_HC_/(cross-bridge) compared to that of WT, whereas E40K did not, indicating that E54K diminished the strong binding of actin and myosin. This phenomenon can be explained by the mechanism in which E40K and E54K not only affect the local structure of Tm, but also affect the overall structure in a different way, leading to two different effects in force generation and cross-bridge kinetics. Electron microscopy and molecular dynamics simulations have shown that Tm mutants D175N and E180G caused a change in the overall structure of Tm [84]. Therefore, we conclude that E40K and E54K may change the overall flexibility of Tm in their own specific ways, thus leading to a DCM phenotype through two different mechanisms.

### Conclusion

We conclude that over-inhibition of the actomyosin interaction by Tm mutants E40K and E54K, which leads to decreased force generation under both high [Ca^2+^] (pCa 4.66) and low [Ca^2+^] conditions (pCa 7.0), is the primary cause of Tm mutation-related DCM pathogenesis.
